# iTRAQ-based analysis of progerin expression reveals mitochondrial dysfunction, reactive oxygen species accumulation and altered proteostasis

**DOI:** 10.1186/s13287-015-0110-5

**Published:** 2015-06-12

**Authors:** Jesús Mateos, Arancha Landeira-Abia, Juan Antonio Fafián-Labora, Pablo Fernández-Pernas, Iván Lesende-Rodríguez, Patricia Fernández-Puente, Mercedes Fernández-Moreno, Aitor Delmiro, Miguel A. Martín, Francisco J. Blanco, María C. Arufe

**Affiliations:** Grupo de Proteómica-ProteoRed/Plataforma PBR2-ISCIII, Servicio de Reumatología, Instituto de Investigación Biomédica de A Coruña (INIBIC), Complexo Hospitalario Universitario de A Coruña (CHUAC), Sergas, Universidade da Coruña, As Xubias, 15006 A Coruña Spain; Cellular Therapy and Medicine Regenerative Group, Department of Medicine, Instituto de Investigación Biomédica de A Coruña (INIBIC), Complexo Hospitalario Universitario de A Coruña (CHUAC), Sergas, Universidade da Coruña, As Xubias, 15006 A Coruña Spain; Rheumatology Division, CIBER-BBN/ISCII, Instituto de Investigación Biomédica de A Coruña INIBIC-Hospital Universitario A Coruña, 15006 A Coruña, Spain; Grupo de Genómica, Instituto de Investigación Biomédica de A Coruña (INIBIC), Complexo Hospitalario Universitario de A Coruña (CHUAC), Sergas, Universidade da Coruña, As Xubias, 15006 A Coruña Spain; Laboratorio de Enfermedades Mitocondriales, Instituto de Investigación Hospital 12 de Octubre (i + 12), Centro de Investigación Biomédica en Red de Enfermedades Raras (CIBERER), U723, Madrid, E-28041 Spain

## Abstract

**Introduction:**

Nuclear accumulation of a mutant form of the nuclear protein Lamin-A, called Progerin (PG) or Lamin AΔ50, occurs in Hutchinson-Gilford Progeria Syndrome (HGPS) or Progeria, an accelerated aging disease. One of the main symptoms of this genetic disorder is a loss of sub-cutaneous fat due to a dramatic lipodystrophy.

**Methods:**

We stably induced the expression of human PG and GFP -Green Fluorescent Protein- as control in 3T3L1 cells using a lentiviral system to study the effect of PG expression in the differentiation capacity of this cell line, one of the most used adipogenic models. Quantitative proteomics (iTRAQ) was done to study the effect of the PG accumulation. Several of the modulated proteins were validated by immunoblotting and real-time PCR. Mitochondrial function was analyzed by measurement of a) the mitochondrial basal activity, b) the superoxide anion production and c) the individual efficiency of the different complex of the respiratory chain.

**Results:**

We found that over-expression PG by lentiviral gene delivery leads to a decrease in the proliferation rate and to defects in adipogenic capacity when compared to the control. Quantitative proteomics analysis showed 181 proteins significantly (p < 0.05) modulated in PG-expressing preadipocytes. Mitochondrial function is impaired in PG-expressing cells. Specifically, we have detected an increase in the activity of the complex I and an overproduction of Superoxide anion. Incubation with Reactive Oxygen Species (ROS) scavenger agents drives to a decrease in autophagic proteolysis as revealed by LC3-II/LC3-I ratio.

**Conclusion:**

PG expression in 3T3L1 cells promotes changes in several Biological Processes, including structure of cytoskeleton, lipid metabolism, calcium regulation, translation, protein folding and energy generation by the mitochondria. Our data strengthen the contribution of ROS accumulation to the premature aging phenotype and establish a link between mitochondrial dysfunction and loss of proteostasis in HGPS.

## Introduction

Mutations in the LMNA gene are the causal agent for a subset of genetic diseases affecting mesoderm tissues called laminopathies [1]. Among these, Hutchinson-Gilford Progeria Syndrome (HGPS) or progeria [2–4] is a fatal disease with a very low incidence characterized by a typical clinical picture of older pathologies [5]. HGPS-affected patients begin to show symptoms of accelerated aging at the age of 2, and typically die at an average age of 13 years, usually due to cardiovascular deficiencies. HGPS is due, in most cases, to the point mutation G608G in the LMNA gene encoding Lamins A and C, major structural components of the nuclear lamina [6, 7]. Although historically thought to be involved only in nuclear structure, roles in replication, chromatin organization and stem cell differentiation have been demonstrated recently for Lamin A [8, 9]. It is also proposed that Lamin A has a role in reorganization of replication and chromatin [10]. Lamin A is initially produced as a precursor, pre-Lamin A, farnesylated at its C-terminus, and processed by the protease Zmpste24/FACE-1 that removes the farnesylated part. In HGPS, the mutation causes the occurrence of a cryptic alternative processing site, generating a truncated isoform, progerin (PG), lacking the recognition site for Zmpste24/FACE-1. Farnesylated PG does not properly process, accumulates in the nuclear envelope, causes structural defects in the nuclear lamina and may be interfering with regulation of the signalling pathway mediated by p16/Rb necessary to maintain the balance between differentiation and proliferation of stem cells in the tissue regeneration process [8]. Finally, several studies showed the importance of accumulation of the farnesylated precursor in the development of the disease [11–13].

The main function of adipose tissue is to store and manage excess energy in the form of triglycerides and to facilitate the liberation and lipolysis in periods of nutritional deficiency or energy demand [14]. The balance between lipid storage and lipolysis is controlled by neuroendocrine signals [15, 16] in response to the nutritional status of the organism. The hypothalamus has been proposed as the central coordinator of this process, integrating the action of circulating hormones and nutrients [17]. In human lipodystrophies, insulin resistance and loss of regenerative potential in the adipose tissue are the main landmarks [18] leading to complications in normal aging and disease. Also, an extremely accelerated lipodystrophia occurs in progeroid syndromes, such as HGPS and other laminopathies [19, 20].

Our purpose for this study was to unravel the underlying mechanism of PG-driven lipodystrophy using quantitative shotgun proteomics (isobaric tags for relative quantification (iTRAQ)) and to determine the molecular pathways modulated by the lentiviral expression of this aberrant form of Lamin A in the 3T3L1 pre-adipocyte cell line, one of the most studied models of adipogenic differentiation [21, 22].

## Methods

### Culture of mouse 3T3L1 pre-adipocytes

3T3L1 cells were kindly donated by María Pardo and Luisa M Seoane (University of Santiago, Spain). Cells were maintained as pre-adipocytes in Dulbecco’s modified Eagle’s medium (DMEM) supplemented with 10 % foetal bovine serum (FBS) and expanded when they reached 70 % confluence.

### Cloning procedure

Full-length human PG and green fluorescent protein (GFP) cDNAs were amplified by PCR from pBABE-puroGFP-PG and pBABE-puroGFP-Lamin A plasmids (Addgene, donated by Tom Misteli), using the oligonucleotides: *Eco*RI-LMNA-forward, CCGGAATTCATGGAGACCCCGTCCCAGCGG; *Bam*HI-LMNA-reverse, CGCGGATCCTTACATGATGCTGCAGTTCTG; *Eco*RI-GFP-forward, CCGGAATTCATGGTGAGCAAGGGCGAG; and *Bam*HI-GFP-reverse, CGCGGATCCTTACTTGTACACCTCGTC. GFP, LMNA and PG were cloned into pLVX-puro (Clontech Laboratories Inc., Mountain View, CA, USA) between the *Eco*RI/*Bam*HI sites following standard cloning procedures.

### Lentiviral production

The Lenti-X™ Lentiviral expression System (Clontech Laboratories Inc.) was used following the manufacturer’s protocol. One day before transfection, 4 × 10^6^ 293 T producer cells were plate on 100 mm plates in penicillin/streptomycin-free DMEM supplemented with 5 % FBS. The following day, two different calcium-phosphate based transfections were performed in duplicate using LVX-GFP-puro and LVX-PG-puro. The cells were incubated overnight with the transfection mixture, then washed with phosphate-buffered saline (PBS) and incubated with 8 ml fresh complete growth medium.

Viral supernatants were collected at 48, 60 and 72 hours following transfection, centrifuged, filtered to remove cell debris and stored at 4 °C until transduction.

### Transduction of 3T3L1 cells

Target cells were plated in 100 mm plates at 6 × 10^6^ cells per plate. After 1 day, the cells were 70 % confluent. The cells were incubated sequentially with the 48-hour, 60-hour and 72-hour viral supernatants for 12 hours. Following the last transduction, the cells were washed and incubated with fresh growth medium to allow puromycin-resistance expression. Two days later, puromycin selection was performed by incubating the cells in growth media supplemented with 1 μg/ml puromycin (Clontech Laboraties Inc.) for 5 days. After selection, transduced cells were washed and allowed to recover in complete media for 2 days.

### Proliferation assay

To calculate the proliferation curve, different numbers of cells (0, 1000, 2000, 4000, 8000 and 16,000) were plated in triplicate in 96-well plates and allowed to adhere for 8 hours. The CellTiter 96® AQueous Non-Radioactive Cell Proliferation Assay (Promega, Madison, WI, USA) was used to measure absorbance at 490 due to the formation of MTS formazan. For the assay, 2000 cells were plated in triplicate in 96-well plates, and the total cell number was calculated at different time points (2, 4, 6 and 10 days) by extrapolating the correspondent absorbance in the proliferation curve.

### Adipogenic differentiation

Untransduced and PG-transduced and GFP-transduced 3T3L1 pre-adipocytes were differentiated in DMEM supplemented with 10 % FBS in chamber slides (Millipore, Billerica, MA, USA). Two days after reaching confluence, the medium was supplemented with 10 μg/ml insulin, 0.5 mM of 3-isobutyl-1-methylxantine and 1 am dexamethasone for 3 days and with 5 μg/ml insulin for a further 6 days.

Adipogenic cultures were stained with Oil red O to determine their adipogenic capacity. For staining, the culture plates were fixed in 10 mM sodium periodate, 2 % paraformaldehyde, 75 mM l-lysine dihydrochloride and 37.5 mM dibasic sodium phosphate (Sigma-Aldrich, St. Louis, MO, USA) at pH 7.4 for 15 minutes at room temperature, then air dried and treated with a filtered solution of 0.3 % Oil red O to stain lipid droplets.

### Protein extraction and preparation procedures

Cells monolayers were grown until a confluence of 70 % in 100 mm plates, and then washed three times with PBS and harvested with a scraper in SDS lysis buffer (20 % glycerol, 500 mM Tris–HCl, pH 6.8 and 10 % sodium dodecyl sulphate). After incubation at 100 °C for 10 minutes and two consecutive cycles of vortexing and sonication, samples were centrifuged at 4 °C for 10 minutes at 11000 × g. Supernatants were then subjected to protein quantification (total protein A 280) in a nanodrop™ 1000 instrument (Thermo Scientific, Waltham, MA, USA). Protein extracts were aliquoted and stored at −80 °C until further analysis.

### iTRAQ labelling

Equal amounts of proteins from PG-3T3L1 and GFP-3T3L1 cells (50 μg) were precipitated with overnight incubation with six volumes of cold acetone at −20 °C, and denatured with 2 % sodium dodecyl sulphate in 1 M triethylammonium bicarbonate (ABSciex, Foster City, CA, USA). The samples were then reduced for 1 hour at 60 °C using 50 mM tris-(2-carboxyethy) phosphine (ABSciex), and cysteine-blocked with 84 mM iodoacetamide (Sigma-Aldrich) at room temperature in the dark for 30 minutes. The injection volumeThe proteins were digested with spectrometry-grade trypsin (Gold Mass, Promega, Madison, WI, USA) at a concentration of 1:50 trypsin/protein for 16 hours at 37 °C. Each peptide solution was labelled for 1.5 hours at room temperature using the iTRAQ reagents (ABSciex) previously reconstituted in 70 μl ethanol, following the manufacturer’s protocol. The samples were labelled with iTRAQ reagents as follows: control GFP, 115; PG: 117. The reaction was stopped by adding deionized water, and the labelled samples were combined. The mixture was desalted using homemade stage tips.

### iTRAQ relative quantification by two-dimensional LC-MALDI-TOF/TOF analysis

In a first step, the desalted peptides (starting amount of digested peptides: 100 μg) were fractionated by basic reversed-phase extraction in a 1200 HPLC system (Agilent, Wilmington, DE, USA). Sixty fractions, each representing a 90-second elution, were collected along a 110-minute gradient. The fractions were combined in 16 different final fractions depending on their peptide complexity (as revealed by lecture absorbance at 214 nm during the basic reversed-phase separation). The combined fractions were subjected to further acidic reversed-phase extraction in a nanoHPLC system (Tempo; ABSciex) into a C18 silica-based column (New Objective, Woburn, MA, USA) with an internal diameter of 300 Ả. The injection volume was 5 μl, and peptides were eluted during a 90-minute gradient at a constant flow rate of 0.35 μl/minute. Eluting peptides were automatically mixed with alpha-cyano at 4 mg/ml in 70 % acetonitrile, 0.1 % trifluoroacetic acid and deposited on a MALDI LC-plate using a SunCollect (SunChrom, Friedrichsdorf,GE) spotter. The 16 different chromatograms, each composed of 350 spots (15 seconds deposition per spot), were then analysed in a 4800 MALDI-TOF/TOF platform (ABSciex). The 4000 series Explorer v.4.2 software was used to generate the spectra and peak list. After manual deposition of mass calibrants, plate model and default calibration of the MALDI plate was carried out with a laser voltage of 3200 kV and 1000 shots/spectrum. Samples were automatically analysed in MS mode with a laser voltage of 3400 kV and 1500 shots/spectrum.

Automated precursor selection was performed using a Job-wide interpretation method (up to 12 precursors/fraction, signal-to-noise lower threshold = 50) excluding trypsin autolytic peptides and other background ions, with a laser voltage of 4200 and 2000 shots/spectrum. The collision induced dissociation (CID) energy range was medium.

Liquid chromatography coupled offline to matrix-assisted laser desorption ionization–time of flight (LC-MALDI-TOF/TOF) data were analysed using ProteinPilot 4.0 software (ABSciex). ProteinPilot search parameters were as follows: sample type, iTRAQ 4-plex; *cys*-alkylation, iodoacetamide; digestion, trypsin; ID focus, biological modifications; database, NCBI RefSeq-release60 (September 2013) with 41,958,567 entries; species filtering, none; search effort, thorough ID and Detection Protein Threshold Unused ProtScore (Conf) > 1.3 (95.0 %). The scoring model was defined by the Paragon algorithm. In the case of the high-complexity samples, the false discovery rate was estimated in less than 1 % by doing the searching in parallel against a decoy database using the ‘PSPEP on’ mode (data not shown).

### Bioinformatics

Biological functional analysis of the different modulated proteins detected by iTRAQ quantification was categorized according to their function, biological process and cellular component, using the String 9.0 software [23].

### Measurement of the production of mitochondrial reactive oxygen species

Cells were cultivated in six-well plates in DMEM with 10 % FBS until reaching 70 % confluence, and then washed and incubated for 1 hour with serum-free DMEM. Incubation with 5 μM MitoSox™ (Invitrogen) was done for 10 minutes at 37 °C following the manufacturer’s instructions. Mitochondrial reactive oxygen species (ROS) production was estimated by flow cytometry in a FACSAria instrument (Benton Dickinson, Oxford, UK). The percentage of positive cells was measured for each condition by fluorescent emission at 580 nm.

### Measurement of the mitochondrial reespiratory chain complex activities

For each condition 10 × 10^6^ cells were collected by trypsinization, washed with PBS, and precipitated at 150 × *g* for 5 minutes at 4 °C. Digitonin-permeabilized pre-adipocyte homogenates (10–50 μl/ml test volume) were used to measure the activities of the respiratory chain enzymes and citrate synthase in a DU-650 spectrophotometer (Beckman Instruments, Palo Alto, CA, USA) as described previously [24, 25].

### Determination of basal mitochondrial respiration

The rate of oxygen consumption (OCR) was determined by direct measurement in a SeaHorse XFp Extracellular Flux Analyzer instrument (Seahorse Bioscience Inc., Billerica, MA, USA). Then 10^4^ cells per well were seeded 24 hours prior to the assay in XF cell culture microplates and incubated at 37 °C with 5 % CO_2_. The next day, the cells were pre-incubated without CO_2_ for 1 hour and the basal OCR was determined following the manufacturer’s instructions.

### Immunoblotting procedures

Western blot analysis was performed on 20 μg total protein extracted from cells in culture, as described previously [26]. The antibodies used were mouse Lamin A + C (Acris Antibodies, Acris Antibodies, Barcelone,ES), rabbit calnexin (Santa Cruz Biotech., Santa Cruz, CA, USA), rabbit nucleolin (NCL; Santa Cruz Biotech.), rabbit cytochrome c oxidase (Amersham, Buckinghamshire, UK), rabbit microtubule-associated protein 1 light chain 3 (LC3; MBL, Nagoya, Japan) and mouse tubulin (Sigma). Ideal concentrations for each antibody were determined empirically. Working concentrations were 1:1000 of the stock solutions. Secondary anti-mouse and anti-rabbit antibodies (Amersham) were used to visualize proteins by the ECL™ Western Blotting Analysis System (GE Healthcare, Amersham Biotechnology, Buckinghamshire, UK). Optimal concentrations for each antibody were determined empirically. Blots were digitized using the LAS 3000 image analyser (GE Healthcare). Densitometry analysis of the band intensities was performed using ImageQuant 5.2 software (GE Healthcare).

### RNA extraction and cDNA synthesis

Total RNA was extracted from cultured cells seeded in six-well plates at a confluence of 70 % using the Trizol L.S. reagent method (Invitrogen). Reverse transcription was done using the Superscript II system (Invitrogen). Then 2 μg total RNA was reverse transcribed in a 20 μl reaction volume containing Superscript II (200 Units), random primers (25 μM) and dNTP (0.5 mM each) at 42 °C for 50 minutes.

### Real-time RT-PCR analysis

The reported sequences of genes for mouse Lmna (forward, TGAGTACAACCTGCGCTCAC; reverse, CTGTGACACTGGAGGGCAGAA), mouse Ncl (forward, AAGGAGTGAAGCCAGCAAAA; reverse, TCCTCCTCAGCCACACTCTT), mouse Canx (forward, GCCCTAGAGACTGCTCCAT; reverse, AAAAAGCCTTGTGCTCCACA), mouse Cox5a (forward, GGAATTGCGTAAAGGGATGA; reverse, CCAAGATGCGAAGACCACTA) and mouse Hprt (forward, TCCCAGCGTCGTGATTAGCGA; reverse, TGGCCTCCCATCTCCTTCATGAC), as housekeeping, were used for primer design. The amplification program consisted of an initial denaturation at 92 °C for 2 minutes followed by 40 cycles at 92 °C for 15 seconds, annealing at 61 °C for 30 seconds and an extension at 72 °C for 15 seconds. Each PCR analysis was done in duplicate, with each set of assays repeated three times. To minimize effects of unequal quantities of starting RNA and to eliminate potential sources of inconsistency, relative expression levels of each gene were normalized to Hprt using the 2^–ΔΔCt^ method [27]. Control experiments contained no reverse transcriptase.

### Statistical analysis

All of the experimental data, with the exception of the iTRAQ relative quantification, are presented as the mean, and error bars represent the standard deviation of the mean. Statistical analysis was carried out with R 3.0 (Project for Statistical Computing) using non-parametric methods.

## Results

### Gene delivery by lentiviral transduction and characterization of cell lines

**Fig. 1:**
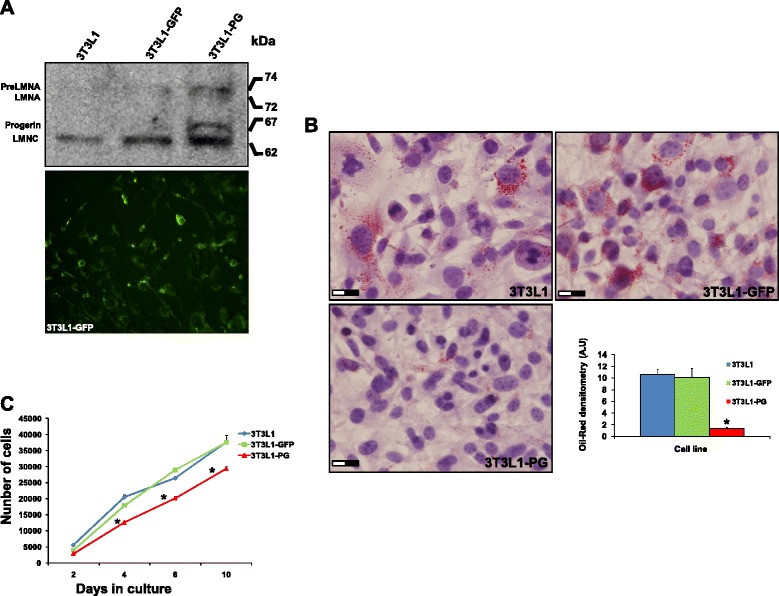
Characterization of green fluorescent protein (GFP)-expressing and progerin (PG)-expressing 3T3L1 cells. **a** Efficient gene delivery of PG and GFP was corroborated by immunoblot and fluorescence microscopy, respectively. **b** Decrease in the proliferation rate of PG-3T3L1 cells when compared with GFP-3T3L1 and non-transduced cells. **c** Defective adipogenic potential of PG-3T3L1 cells when compared with GFP-3T3L1 and non-transduced cells, as revealed by Oil-red staining. *Significance (*p* <0.05) using the Kruskal–Wallis non-parametric test. Scale bar = 20 μm

Efficient gene delivery of PG was checked by immunoblotting using an anti-LMNA/C antibody. As shown in Fig. 1a, PG-lentiviral transduction of 3T3L1 pre-adipocytes drives the production of an intermediate molecular weight isoform with a similar size as human PG, as well as an abnormal accumulation of wild-type Lamin A as described previously [28, 29]. The efficiency of the transduction was estimated in more than 80 % by fluorescence microscopy. Proliferation capacity was checked in the three cell lines, showing a significant decrease (*p* <0.05) in the proliferation rate of PG-3T3L1 cells when compared with non-transduced and control GFP-3T3L1 cells. Adipogenic capacity of the cell lines was tested by directed differentiation in adipogenic medium for 7 days followed by Oil red staining and haematoxylin and eosin counterstaining (Fig. 1c). PG-3T3L1 cells show a defective adipogenic capacity when compared with non-transduced and control GFP-3T3L1 cells. Oil red densitometry demonstrates a significant (*p* <0.05) decrease in the total area of the lipid droplets (Fig. 1c).

### iTRAQ relative quantification

**Fig. 2:**
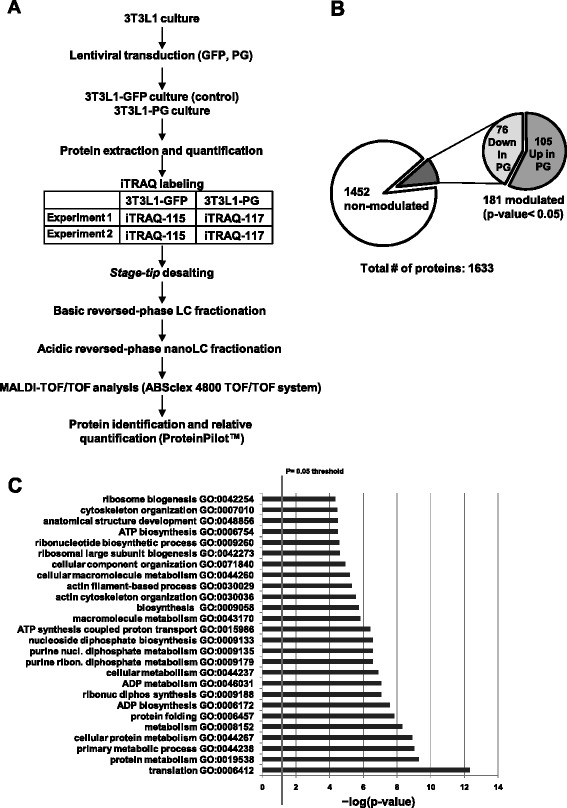
Isobaric tags for relative quantification (iTRAQ) of modulated proteins in 3T3L1-PG cells. **a** Workflow followed for the relative quantification of modulated proteins in PG-3T3L1 cells. **b** Summary of the protein identification and relative quantification. **c** Significantly modulated biological processes after String 9.0 analysis of the modulated individual proteins. *GFP* green fluorescent protein, *GO* gene ontology, *LC-MALDI-TOF* liquid chromatography coupled offline to matrix-assisted laser desorption ionization–time of flight, *PG* progerin

A summary of the workflow followed for iTRAQ relative quantification of modulated proteins in PG-3T3L1 cells versus GFP-3T3L1 cells is shown in Fig. 2a. A total of 1633 proteins were identified after grouping; 76 of them were significantly decreased whereas 105 were increased in PG-3T3L1 versus control (Fig. 2b). Proteins were considered significantly modulated if the 117/115 ratio was higher than 1.4 or lower than 0.7, always with *p* <0.05. Only proteins presenting similar significant ratios in both iTRAQ experiments 1 and 2 were considered modulated. The detailed list of modulated proteins classified by principal biological process is presented in Table 1.

**Table 1:** Modulated proteins (*p* <0.05) in PG-3T3L1 cells classified according to their principal biological process

Accession number	Name	Total number of peptides	iTRAQ 1 ratio PG/control	iTRAQ 2 ratio PG/control
Actin cytoskeleton organization				
gi|157951604	Adenylyl cyclase-associated protein 1	9	1.80	1.81
gi|6680924	cofilin-1	22	2.63	2.63
gi|6681069	Cysteine and glycine-rich protein 1	9	0.41	0.44
gi|62526118	Cytoskeleton-associated protein 4	22	6.02	5.81
gi|125347376	Filamin-A	123	0.06	0.06
gi|145966915	Filamin-B	89	0.18	0.22
gi|124487139	Filamin-C	51	0.42	0.44
gi|130488506	Four and a half LIM domains protein 3	8	0.39	0.34
gi|61657921	Kinesin-1 heavy chain	14	0.49	0.44
gi|6755040	Profilin-1	19	2.90	2.94
gi|10946578	Thymosin beta-4	15	0.42	0.44
gi|47894398	Tropomyosin alpha-4 chain	51	0.57	0.60
gi|31982755	Vimentin	101	27.8	29.5
Alternative splicing				
gi|85060507	Heterogeneous nuclear ribonucleoprotein A1	27	6.94	8.24
gi|21313308	Heterogeneous nuclear ribonucleoprotein M	17	2.70	2.85
gi|283436178	Heterogeneous nuclear ribonucleoproteins C1/C2	10	1.82	1.85
gi|6678143	Lupus La protein homolog	9	1.57	1.89
gi|31560656	Polyadenylate-binding protein 1	24	1.55	1.88
gi|126157504	Serine/arginine repetitive matrix protein 2	12	0.33	0.35
gi|34328400	Serine/arginine-rich splicing factor 1 isoform 1	12	0.58	0.63
gi|153791358	Splicing factor 3B subunit 1	7	0.50	0.52
gi|268837785	Splicing factor 3b, subunit 2	16	1.59	1.62
gi|23956214	Splicing factor, proline- and glutamine-rich	22	1.48	1.45
ATP metabolism				
gi|6680748	ATP synthase subunit alpha	21	2.53	2.48
gi|31980648	ATP synthase subunit beta	32	5.89	5,84
gi|7949005	ATP synthase-coupling factor 6	8	0.35	0.38
gi|150456419	ATP-dependent RNA helicase A	10	0.52	0.45
gi|134288917	Cytoplasmic dynein 1 heavy chain 1	16	0.48	0.53
Calcium-mediated signalling				
gi|6996913	Annexin A2	28	1.89	1.72
gi|6753060	Annexin A5	26	6.01	6.47
gi|21704156	Caldesmon 1	31	2.17	2.49
gi|6753244	Calmodulin	22	0.31	0.31
gi|6671664	Calnexin	14	1.62	2.44
gi|21312564	Calponin-3	15	2.01	1.79
gi|6680836	Calreticulin precursor	31	3.37	3,98
gi|31981086	EF-hand domain-containing protein D2	6	0.39	0.40
gi|6679465	Glucosidase 2 subunit beta precursor	9	0.59	0.69
gi|33620739	Myosin light polypeptide 6	24	0.69	0.39
gi|6677833	Protein S100-A10	11	0.28	0.31
gi|6677691	Reticulocalbin-1 precursor	10	0.50	0.69
gi|114205428	Reticulocalbin-2 precursor	12	0.48	0.45
gi|188035858	Reticulocalbin-3 precursor	13	0.41	0.54
gi|295054266	Spectrin alpha chain, brain isoform 2	80	0.60	0.58
gi|117938332	Spectrin beta chain, brain 1 isoform 1	45	0.50	0.43
Cell adhesion				
gi|61743961	AHNAK nucleoprotein isoform 1	265	0.63	0.62
gi|6754508	LIM and SH3 domain protein 1	15	0.55	0.59
gi|225543161	Lipoma-preferred partner homolog isoform 1	18	0.55	0.55
gi|70778915	Moesin	41	3.31	2.80
gi|33598964	Myosin-10	42	0.18	0.19
gi|114326446	Myosin-9 isoform 1	127	0.05	0.04
gi|254675244	Plectin isoform 1	151	0.31	0.56
gi|377835925	Protein AHNAK2-like	16	0.78	0.54
gi|227116327	Talin-1	42	0.25	0.24
gi|6678347	Thy-1 membrane glycoprotein preproprotein	5	4.15	3.93
gi|6756085	Zyxin	19	0.52	0.55
gi|114326497	Laminin subunit beta-1	5	0.34	0.37
gi|31982030	Rho GDP-dissociation inhibitor 1	10	2.60	2.49
DNA replication				
gi|6679299	Prohibitin	9	1.82	2.39
gi|7242171	Proliferating cell nuclear antigen	10	1.73	1.78
Extracellular matrix organization				
gi|34328108	Collagen alpha-1(I) chain precursor	56	0.66	0.49
gi|111120329	Collagen alpha-2(I) chain precursor	53	0.67	0.68
gi|33859580	Galectin-3	12	1.96	1.81
gi|33859596	Prolyl 4-hydroxylase subunit alpha-1 precursor	14	1.89	1.89
Glycolysis				
gi|6679937	Glyceraldehyde-3-phosphate dehydrogenase	33	25.5	23.1
gi|70778976	Phosphoglycerate kinase 1	40	3.37	3.18
gi|226958349	Triosephosphate isomerase	20	2.58	2.53
gi|70794816	Alpha enolase	55	16.8	20.2
Golgi apparatus function				
gi|254750698	Nucleobindin-1 isoform 1 precursor	17	0.53	0.54
gi|194440700	Nucleobindin-2 isoform 1 precursor	5	0.53	0.54
Lipid metabolism				
gi|6681137	Acyl-CoA-binding protein isoform 2	10	0.29	0.29
gi|63999380	Alpha-2-macroglobulin receptor-associated	4	0.40	0.42
gi|313151222	ATP-citrate synthase isoform 1	10	0.46	0.53
gi|93102409	Fatty acid synthase	8	0.55	0.55
gi|225735657	Sulphated glycoprotein 1 isoform F preproprotein	9	5.49	5.24
Lipid transport				
gi|19527028	Vigilin	15	1.52	1.47
gi|38198665	NSFL1 cofactor p47	13	0.69	0.64
Nuclear structure				
gi|162287370	Prelamin-A/C isoform A	47	4.62	4.79
gi|7110705	Prothymosin alpha	7	0.42	0.43
Nucleosome assembly				
gi|7949045	Histone H2A.Z	6	1.73	1.49
gi|13591862	Protein SET isoform 1	21	0.22	0.27
Nucleotide biosynthesis				
gi|209862992	Dihydropyrimidinase-related protein 3 isoform 1	18	1.95	1.75
gi|377835587	inosine-5′-monophosphate dehydrogenase 2-like	9	1.47	1.68
Oxidation–reduction process				
gi|31542438	Cytochrome b5 type B precursor	6	3.05	3.20
gi|112181182	Cytochrome c oxidase subunit 5A	12	0.35	0.28
gi|6681095	Cytochrome c, somatic	9	0.44	0.41
gi|112293264	Protein disulphide-isomerase A3 precursor	49	6.03	5.80
gi|86198316	Protein disulphide-isomerase A4 precursor	27	1.55	1.61
gi|42415475	Protein disulphide-isomerase precursor	44	1.86	1.87
gi|6755911	Thioredoxin	10	0.47	0.47
Protein catabolic process				
gi|6679501	26S protease regulatory subunit 4	15	2.21	2.59
gi|228008337	26S protease regulatory subunit 6A	16	1.97	1.93
gi|124248577	26S protease regulatory subunit 6B	12	2.27	2.37
gi|19882201	26S proteasome non-ATPase regulatory subunit	9	0.54	0.52
gi|6755212	Proteasome activator complex subunit 1	12	0.60	0.58
Protein folding and stress response				
gi|6680309	10 kDa heat shock protein, mitochondrial	8	0.34	0.34
gi|183396771	60 kDa heat shock protein, mitochondrial	46	23.9	13.9
gi|115270960	BAG family molecular chaperone regulator 3	9	0.57	0.59
gi|6755863	Endoplasmin precursor	42	6.42	7.51
gi|31981690	Heat shock cognate 71 kDa protein	68	14.4	19.9
gi|6754254	Heat shock protein HSP 90-alpha	44	2.22	2.26
gi|40556608	Heat shock protein HSP 90-beta	48	2.55	2.46
gi|27229055	Huntingtin-interacting protein K	5	0.40	0.42
gi|161353506	Serpin H1 precursor	22	2.44	2.39
gi|162461907	Stress-70 protein, mitochondrial	52	2.05	2.04
gi|126521835	T-complex protein 1 subunit beta	18	1.71	1.68
gi|6671702	T-complex protein 1 subunit epsilon	16	1.59	1.51
gi|6753324	T-complex protein 1 subunit zeta	11	1.66	1.75
Protein transport				
gi|28077049	Charged multivesicular body protein 4b	9	0.51	0.51
gi|51491845	Clathrin heavy chain 1	18	0.52	0.48
gi|122939198	Clathrin light chain A isoform c	7	0.44	0.45
gi|88014720	Importin subunit beta-1	9	0.41	0.42
gi|124486712	Ribosome-binding protein 1 isoform a	20	0.60	0.65
Regulation of apoptosis and cell death				
gi|226874906	14-3-3 protein epsilon	13	2.01	1.62
gi|6756041	14-3-3 protein zeta/delta isoform 1	15	2.55	2.61
gi|6678682	Galectin-1	31	0.70	0.59
gi|329755243	Gelsolin isoform 2	22	1.85	1.89
gi|165932375	Plasminogen activator-inhibitor 1 RNA-binding protein	31	0.67	0.68
gi|9790259	Programmed cell death protein 5	8	0.49	0.35
gi|6755963	Voltage-dependent anion-selective channel protein 1	15	2.53	2.41
Regulation of canonical Wnt signalling				
gi|6679641	Emerin	8	2.87	3.42
gi|7305075	ras GTPase-activating protein-binding protein 1	16	2.25	1.99
gi|158854016	S-phase kinase-associated protein 1	7	0.50	0.50
Regulation of cell proliferation				
gi|124517663	Annexin A1	28	2.44	1.88
gi|110625813	Astrocyte-derived neurotrophic factor precursor	11	0.47	0.47
gi|28461294	Protein CDV3 isoform b	8	0.52	0.57
gi|356640163	Serine hydroxymethyltransferase, mitochondrial	17	1.90	1.72
gi|6678483	Ubiquitin-like modifier-activating enzyme 1	15	0.59	0.58
Transcription				
gi|188497724	Hepatoma-derived growth factor	12	0.55	0.50
gi|84875537	Nucleolin	33	3.60	4.17
gi|6679567	Polymerase I and transcript release factor	17	2.23	2.65
gi|13386026	UPF0568 protein C14orf166 homolog	9	0.60	0.60
Translation and ribosome assembly				
gi|309266241	60S ribosomal protein L29-like	4	1.90	1.90
gi|309263511	60S ribosomal protein L32-like	6	7.27	9.17
gi|21426889	40S ribosomal protein S11	11	2.99	2.84
gi|13386034	40S ribosomal protein S13	10	4.74	6.81
gi|6677799	40S ribosomal protein S15	11	2.26	1.82
gi|12963511	40S ribosomal protein S19	10	2.92	3.02
gi|18087805	40S ribosomal protein S2	14	2.75	2.88
gi|13195604	40S ribosomal protein S23	7	5.35	4.83
gi|6755372	40S ribosomal protein S3	16	1.64	1.65
gi|254553321	40S ribosomal protein S3a	13	2.75	2.65
gi|6677805	40S ribosomal protein S4, X isoform	14	7.60	4.25
gi|6677813	40S ribosomal protein S8	12	5.30	5.39
gi|33504483	40S ribosomal protein S9	11	4.41	4.01
gi|6671569	60S acidic ribosomal protein P0	10	1.40	1.56
gi|83745120	60S acidic ribosomal protein P2	14	0.31	0.30
gi|16418339	60S ribosomal protein L10	11	3.04	3.81
gi|31981945	60S ribosomal protein L13a	9	3.96	3.00
gi|13385036	60S ribosomal protein L15	7	4.40	3.52
gi|83699424	60S ribosomal protein L18	5	3.03	3.22
gi|58037465	60S ribosomal protein L18a	6	2.99	2.92
gi|226958657	60S ribosomal protein L19 isoform 2	7	4.35	4.19
gi|18250296	60S ribosomal protein L24	6	4.45	4.11
gi|6677777	60S ribosomal protein L26	7	3.80	3.31
gi|8567400	60S ribosomal protein L27	9	5.65	5.99
gi|255308899	60S ribosomal protein L3	20	3.47	3.23
gi|94386224	60S ribosomal protein L36-like	4	9.03	8.15
gi|30794450	60S ribosomal protein L4	20	5.75	5.20
gi|23956082	60S ribosomal protein L5	10	2.04	1.32
gi|84662736	60S ribosomal protein L6	11	7.24	8.24
gi|31981515	60S ribosomal protein L7	9	5.65	6.23
gi|7305443	60S ribosomal protein L7a	9	6.30	6.42
gi|6755358	60S ribosomal protein L8	7	8.87	6.51
gi|14149647	60S ribosomal protein L9	9	1.81	2.29
gi|254540168	78 kDa glucose-regulated protein precursor	49	5.20	4.12
gi|82617575	Bifunctional glutamate/proline--tRNA ligase	6	0.43	0.44
gi|126032329	Elongation factor 1-alpha 1	33	2.48	2.63
gi|31980922	Elongation factor 1-beta	16	0.39	0.30
gi|33859482	Elongation factor 2	44	0.32	0.50
gi|21450625	Eukaryotic initiation factor 4A-I isoform 1	23	2.29	1.96
gi|146219837	Eukaryotic translation initiation factor 3	19	0.50	0.63
gi|365906249	Eukaryotic translation initiation factor 3	8	0.49	0.32
gi|167234372	Eukaryotic translation initiation factor 4B	23	0.59	0.60
gi|31712036	Eukaryotic translation initiation factor 5A-1	26	0.53	0.54
gi|94367038	40S ribosomal protein S6-like isoform 2	6	5.49	5.50
gi|309264022	40S ribosomal protein SA-like	16	3.02	4.32
gi|149251177	60S ribosomal protein L13-like	8	4.11	3.18
gi|377837258	60S ribosomal protein L23-like	8	3.83	3.84
gi|63572172	60S ribosomal protein L27a-like	4	6.99	5.21
gi|37497112	Putative RNA-binding protein 3 isoform 1	8	0.59	0.55
gi|31982373	rRNA 2′-O-methyltransferase fibrillarin	8	2.15	2.07

*iTRAQ* isobaric tags for relative quantification, *PG* progerin

The list of modulated proteins was used to unravel, by String 9.0 analysis, the molecular pathways altered in PG-3T3L1 cells (Fig. 2c). Among the most important processes determined are those related to translation (gene ontology (GO) process: 0006412), ribosome biogenesis (GO process: 0042273) and protein folding (GO process: 0006457), but also metabolism (GO process: 0008152), energy generation by the mitochondria (GO process: 0006754) and structure of the actin cytoskeleton (GO process: 0030036).

### Immunoblotting and real-time PCR validation of selected modulated proteins

**Fig. 3:**
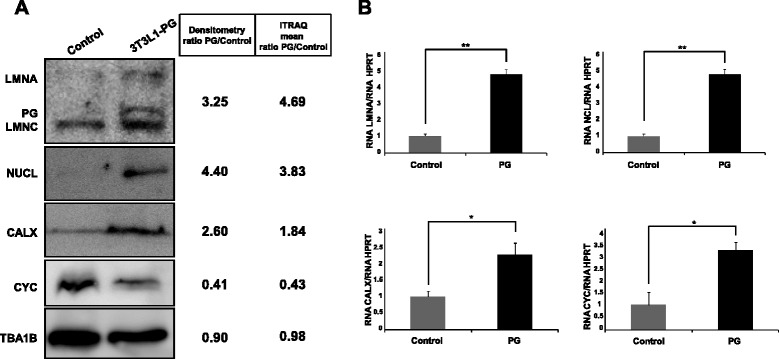
Immunoblotting and RT-PCR verification of selected proteins. **a** Western blotting analysis confirms the modulation of Lamin A, calnexin (CALX), nucleolin (NCL), and cytochrome c (CYC). Loading of equal amount of total protein was tested by Ponceau red staining (not shown) and α-tubulin immunoblotting. **b** Real-time PCR analysis of the selected proteins normalized against Hprt gene levels. *Significance (*p* <0.05) using the Kruskal–Wallis non-parametric test. **Significance (*p* <0.01) using the Kruskal–Wallis non-parametric test. *iTRAQ* isobaric tags for relative quantification, *PG* progerin

Orthogonal validation of iTRAQ results by western blotting (Fig. 3) demonstrated in the first place that Lamin A (ratio PG/control = 4.69) is actually accumulated in PG-3T3L1. Besides, immunoblotting of NCL and calnexin, both accumulated in PG-3T3L1, show similar densitometry ratios (4.40 and 2.60) to those obtained by iTRAQ quantification (3.83 and 1.84, respectively). Finally, cytochrome c is decreased in PG-3T3L1 cells with a similar densitometry ratio (0.41) to that obtained by iTRAQ quantification (0.43). Real-time PCR data are in concordance with the proteomics data, with the exception of cytochrome c. This gene is overexpressed in PG-3T3L1 cells when compared with control.

### Study of the mitochondrial function in PG-3T3L1 cells

**Fig. 4:**
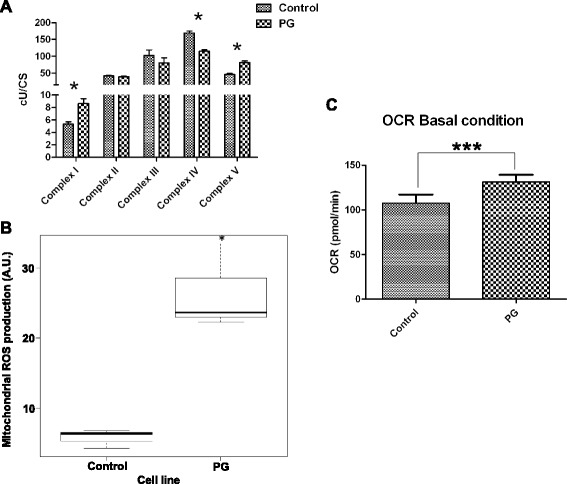
Mitochondrial dysfunction and ROS overproduction in 3T3L1-PG cells. **a** Measurement of the mitochondrial complex activity in digitonin-permeabilized cells demonstrates significant changes in complexes I, IV and V. **b** PG-3T3L1 cells show a significant increase in the mitochondrial ROS production, as revealed by MitoSox™ analysis. **c** Measurement of the rate of oxygen consumption (OCR) indicates that PG-3T3L1 cells have an increased mitochondrial basal activity. *Significance (*p* <0.05) using the Kruskal–Wallis non-parametric test. ***Significance (*p* <0.001) using the Kruskal–Wallis non-parametric test. Scale bar = 10 μm. *CS* citrate synthase, *PG* progerin

Since iTRAQ data suggest a dysfunction of the mitochondrial activity and a disorganization of the nuclear–mitochondrial cytoskeleton network we decided to determine the integrity of the mitochondrial network in PG-3T3L1 cells. First spectrophotometric analysis of the mitochondrial respiratory chain demonstrates significant differences in the activity of electron carrier complex I, IV and V (Fig. 4a). The mitochondrial superoxide anion was determined by flow-cytometry measurement of MitoSox™-treated cells, revealing that PG-expressing cells show a significant (*p* <0.05) increase in superoxide anion production (Fig. 4b). Furthermore, the OCR, a parameter that is proportional to mitochondrial basal activity, is also significantly (*p* <0.001) increased in PG-3T3L1 cells (Fig. 4c).

### Reduction of ROS levels with ROS scavengers and the effect of autophagy

**Fig. 5:**
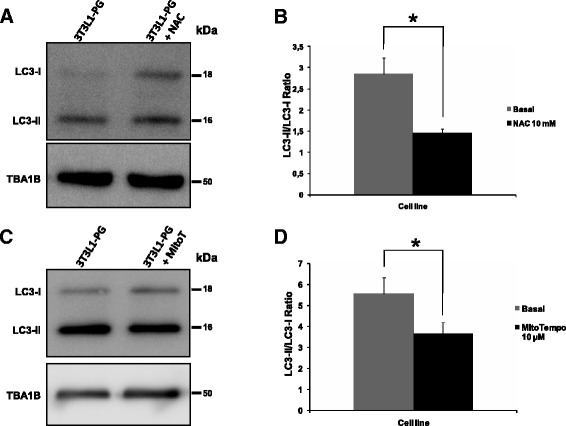
Effect of ROS scavengers on the autophagic proteolysis in PG-3T3L1 cells. **a**, **b** Western blot analysis of LC3 demonstrates a significant reduction in the LC3-II/LC3-I ratio in PG-3T3L1 cells incubated with 10 mM *N*-acetyl-cysteine (NAC) for 1 hour when compared with basal conditions. **c**, **d** Western-blot analysis of LC3 demonstrates a significant reduction in the LC3-II/LC3-I ratio in PG-3T3L1 cells incubated with 10 μM MitoTempo for 1 hour when compared with basal conditions. *LC3* light chain 3, *PG* progerin

Incubation of PG-3T3L1 with a general ROS scavenger (*N*-acetyl-cysteine (NAC) 10 mM for 1 hour; Fig. 5a, b) or a superoxide-specific quencher (MitoTempo 10 μM for 1 hour; Fig. 5c, d) results in a significant (*p* <0.05) reduction of the autophagic marker LC3-II/LC3-I ratio, indicating a decrease in autophagic proteolysis.

## Discussion

Aging is defined as the accumulation of diverse deleterious changes occurring in cells and tissues with advancing age that are responsible for the increased risk of disease and death [30]. Aging is now considered to be a very complex, multi-factorial process [31]. Several aging theories have been proposed, but to date no single theory explains all of the major age-related physiological changes. Among those theories, the role of mitochondrial dysfunction and free radical accumulation in aging and disease has gained strength in recent years [32–35], in part owing to the satisfactory effects exerted by antioxidant compounds in in-vitro studies [36] and therapy [37–39].

Premature aging syndromes are used as models to understand molecular causes of aging-related changes. Among these diseases, HGPS is one of the most studied, although to date few proteomics studies have tried to deep into the mechanisms triggered by the accumulation of the causal agent (PG). Peinado et al. [40] demonstrated using a differential in gel electrophoresis (DIGE) approach that mitochondrial dysfunction and disorganization of the cytoskeleton were characteristic of the adipose tissue in *ZMPSTE24* null mice, which mimic some but not all of the symptoms associated with HGPS-derived cells. Recently, similar results have been found in HGPS cells [41] following a stable isotopic labeling approach. To examine by quantitative proteomics (iTRAQ) the effect of the impairment of the expression of Lamin A, we used a lentiviral system to efficiently deliver PG expression in 3T3L1 pre-adipocytes. The efficient expression of PG in PG-transduced 3T3L1 cells was corroborated by immunoblotting (Fig. 1a). These cells present a decrease in both proliferative capacity and adipogenic potential (Fig. 1b, c), which is in agreement with previous results [29]. Since GFP-3T3L1 cells are similar to non-transduced 3T3L1 in terms of lamin expression, adipogenic capacity and proliferative rate, we decided to use that cell line as a control for the iTRAQ labelling and subsequent validations. A total of 1633 different proteins were quantified, and 181 of them were significantly modulated in PG-expressing cells versus control. To our knowledge, our study represents the first iTRAQ-based proteomic analysis on the effect of PG expression to date.

### Quantitative proteomics data suggest massive modulation of cytoskeleton organization, cell proliferation and protein synthesis processes

String 9.0 analysis demonstrate that expression of PG in 3T3L1 cells promotes changes in protein translation, energy generation by mitochondria, protein folding, response to stress, lipid metabolism and cytoskeleton organization.

Expression of PG in 3T3L1 cells promotes modulation of a large set of regulators and structural components of the cytoskeleton, mainly those involved in the proper maintenance of the filamentous network connecting nucleus, endoplasmic reticulum and mitochondria. Of special interest we have detected upregulation of vimentin (ratio PG/control = 27.28) and cytoskeleton-associated protein 4 (ratio PG/control = 5.9), and downregulation of filamins A (ratio PG/control = 0.06), B (ratio PG/control = 0.18) and C (ratio PG/control = 0.43). Modulation of vimentin has been described previously in Zmpste24^−/−^ mice [40] and HGPS-derived cell lines [41].

Calcium ions are signalling molecules in living cells and play essential roles in cellular processes such as cell proliferation, enzyme activity, muscle contraction, cellular motility, maintenance of the structural integrity of the cytoskeleton and membrane transport, among others [42]. Our proteomics data show modulation of a set of proteins involved in calcium-mediated signalling, including calmodulin (ratio PG/control = 0.31), caldesmon 1 (ratio PG/control = 2.29), calnexin (ratio PG/control = 1.83) and calreticulin ratio (PG/control = 3.73). Of special interest, calnexin is part of the quality control machinery that holds back improperly folded proteins in the endoplasmic reticulum [43]. Western blot and real-time PCR validation (Fig. 3) confirms the increase of calnexin in PG-3T3L1 cells, suggesting that protein misfolding is enhanced by PG expression, a phenomenon described previously in biological [44, 45] and PG-related [46] aging.

It is now widely accepted that PG accumulation modulates molecular pathways such as mTOR, pRB and Wnt signalling cascades, driving alterations in the balance between apoptosis, cell differentiation and cell proliferation [8, 9, 47]. In our model we have detected modulation of proteins belonging to those three processes. For instance, voltage-dependent anion-selective channel protein 1, involved in the formation of apoptotic pores in the mitochondria [48], is upregulated in PG-3T3L1 cells (ratio PG/control = 2.53), as well as emerin (ratio PG/control = 3.31). This protein interacts with LAP2α and links nuclear Lamin A and actin cytoskeleton, negatively modulating accumulation of beta-catenin in the nucleus [49, 50].

Proteomic data strongly suggest a massive modulation of all molecular processes that culminate in protein synthesis and function. Of special interest is the modulation detected on NCL, the major component of the nucleolus and a repressor of the transcription [51]. This protein is upregulated in PG-expressing 3T3L1 cells (ratio PG/control = 3.84). Western blot validation confirms the quantitative proteomics result, as well as real-time PCR analysis. Interestingly, NCL associates with chromatin and is a nucleolar marker of several pathological conditions, ranging from cancer to autoimmune diseases [52]. Protein SET, which mediates histone acetylation and nucleosome assembly, appears dramatically decreased in PG-expressing cells (ratio PG/control = 0.24). Also, a large set of structural ribosomal proteins and translation initiation factors appear to be modulated in our proteomic screening, in agreement with previous data from Rivera-Torres et al. [41], indicating that PG expression exerts a dramatic alteration in protein synthesis and function.

Quantitative proteomic data suggest that PG delivery induces defects on de-novo lipid synthesis and energy storage. Three main proteins are decreased in PG-3T3L1 cells. ATP-citrate synthase (ratio PG/control =0.49), fatty acid synthase (ratio PG/control = 0.55) and acyl-CoA-binding protein (ratio PG/control =0.29). All of them are part of the cellular machinery responsible for the condensation of acetyl CoA and malonyl CoA and the formation of medium and long-chain fatty acids and triacylglycerides that are the main energy repository in the cells [53]. Furthermore, inhibition of ATP-citrate synthase has been recently linked to a decrease in proliferation rate [54], which has been detected in our model (Fig. 1) and is a typical characteristic of PG-expressing cells. Our data agree with previous results from other PG-expression models [28, 40].

### Mitochondrial dysfunction and imbalance in stress response are linked to loss of proteostasis and increase of autophagy in progerin-expressing cells

Since energy storage and lipogenesis are modulated in our model of PG overexpression, it is plausible, a priori, to assume that ATP generation by the mitochondria and oxidation–reduction processes could be also affected. iTRAQ analysis demonstrated modulation of several components of the mitochondrial respiratory chain, especially complex IV and complex V. The complex III–complex IV electron carrier cytochrome c (ratio PG/control = 0.43) and the complex IV component cytochrome c oxidase (ratio PG/control = 0.32) are significantly decreased in PG-3T3L1 cells. Cytochrome c decrease has been validated by immunoblotting (Fig. 3) and is in agreement with previous results [41]. However, real-time PCR analysis of cytochrome c shows an increase in the expression of this gene in PG-3T3L1 cells.

We asked whether this modulation of several components of the mitochondrial respiratory chain detected in PG-3T3L1 cells by quantitative proteomics drives uncoupling of the electron transport chain. Measurement of the MRC complex activities demonstrates significant differences in the activity of complex IV and V, which agrees with previous results [41], but also complex I (Fig. 5a). The activity of this complex is twice as high in PG-3T3L1 when compared with control. Complex I is the main contributor for the generation of mitochondrial ROS and specifically the superoxide anion [55, 56], which has been proposed as one of the factors responsible for several mitochondrial pathologies [57] and plays a central role in the free radical theory of aging [31]. We have found a significantly overproduction of mitochondrial superoxide production in PG-3T3L1 cells, as revealed by MitoSox measurement (Fig. 4b).

Heat shock proteins (hsp) and chaperones are responsible for monitoring the quality of the proteins, to maintain the homeostasis of the proteome (proteostasis) and to derive incorrect isoforms for ATP-dependent degradation by the proteasome, and are upregulated in living cells in response to environmental stress conditions. We have found a dramatic increase of this group of proteins in PG-3T3L1 cells, indicating an activation of chaperone-mediated autophagy (CMA), a process recently described in age-related pathologies [58, 59]. The most obvious examples are hsp60 (ratio PG/control = 18.53), heat shock cognate 71 kDa protein (ratio PG/control =16.59) and endoplasmin or GRP94 (ratio PG/control =6.91). Hsp60 is one of the central components that regulate proteostasis during redox imbalance [60], and is upregulated in stress and disease. Heat shock cognate 71 kDa protein acts as a repressor at the transcriptional level [61]. Finally, GRP94 is a marker of endoplasmic reticulum-mediated degradation of proteins and has been recently involved in regulation of the canonical *Wnt* signalling pathway [62]. The loss of proteostasis could explain the apparent discordance between the proteomics and genomics data for cytochrome c. The elevated levels of mitochondrial ROS could promote an increase in the misfolding of the components of the respiratory chain, driving an abnormal rate of protein degradation. In any case, further investigation is necessary to demonstrate this hypothesis.

Previously, an increase in autophagic proteolysis has been reported in the progeroid phenotype [63, 64], suggesting that chronic activation of an, a priori, pro-survival cellular mechanism can turn into a harmful and detrimental process. We therefore asked whether a reduction of ROS cellular levels could promote a modulation of this process. Incubation of the PG-3T3L1 cells with a general ROS scavenger (NAC 10 mM) or a superoxide anion-specific scavenger (MitoTempo 10 μM) exerts a significant (*p* <0.05) reduction of the LC3-II/LC3-I ratio (Fig. 5), pointing to a reduction in the autophagic proteolysis in accordance with the growing evidence supporting the key role of ROS in the aging process.

## Conclusions

**Fig. 6:**
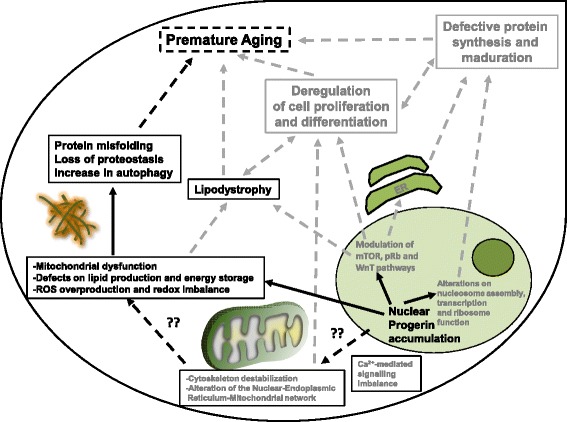
Model representing the main cellular events triggered by lentiviral-driven PG nuclear accumulation and their contribution to the premature aging. PG accumulation leads to modulation of mTOR and WnT pathways, alteration in the protein synthesis machinery and mitochondrial dysfunction and reactive oxygen species (ROS) overproduction, ultimately contributing to the premature aging phenotype

Our study represents one further step in the achievement of understanding the aging process. With our results, but also taking into account the results of excellent studies made by other research groups in recent years, we have tried to build a global scheme with a compilation of the main cellular events that leads to premature aging in HGPS driven by PG accumulation (Fig. 6). In the present work, we have demonstrated that PG expression promotes mitochondrial dysfunction and ROS overproduction. These events are, at least in part, the cause of an increase in protein misfolding and autophagic proteolysis leading, ultimately, to a global loss of proteostasis that partially contributes to the premature aging process. We have also demonstrated that treatment of the cells with antioxidant compounds reduces the autophagic phenotype, thus supporting the growing evidence indicating the importance of this kind of compound as candidates for therapeutics in aging-related diseases.

## Abbreviations

CMA: Chaperone-mediated autophagy
DIGE: Differential in gel electrophoresis
DMEM: Dulbecco’s modified Eagle’s medium
FBS: Foetal bovine serum
GFP: Green fluorescent protein
GO: Gene ontology
HGPS: Hutchinson-Gilford Progeroid Syndrome
hsp: Heat shock protein
iTRAQ: Isobaric tags for relative quantification
LC3: Light chain 3
LC-MALDI-TOF/TOF: liquid chromatography coupled offline to matrix-assisted laser desorption ionization–time of flight
LMNA: Lamin A
NAC: *N*-acetyl-cysteine
NCL: Nucleolin
OCR: Rate of oxygen consumption
PBS: Phosphate-buffered saline
PG: Progerin
ROS: Reactive oxygen species
